# Protective effects of a commercial total flavonoid preparation from *Puerariae Lobatae Radix* on exhaustive exercise–induced liver injury in mice

**DOI:** 10.3389/fphar.2026.1823011

**Published:** 2026-05-20

**Authors:** Zhenmao Zhou, Fan Yang, Chengkun Zhao, Xiangchen Qiu, Ding Yuan

**Affiliations:** 1 School of Medical Humanities, Anhui Medical University, Hefei, Anhui, China; 2 The First Clinical College, Anhui Medical University, Hefei, Anhui, China

**Keywords:** commercial total flavonoid preparation, exhaustive exercise, inflammation, liver injury, oxidative stress, *Puerariae Lobatae Radix*

## Abstract

**Objective:**

This study investigated the protective effects of a commercial total flavonoid preparation derived from *Puerariae Lobatae Radix* on exhaustive exercise–induced liver injury in mice, with a focus on oxidative stress, inflammatory responses, and NF-κB–related signaling.

**Methods:**

A mouse model of exhaustive exercise–induced liver injury was established by exhaustive swimming. Mice were assigned to a normal control group, an exercise model group, a silymarin-treated group, and low-, medium-, and high-dose total flavonoid preparation groups. Liver function, histopathological alterations, oxidative stress–related parameters, inflammatory mediators, and NF-κB–related signaling proteins were evaluated after 4 weeks of treatment.

**Results:**

Exhaustive exercise induced marked liver injury, as evidenced by elevated serum ALT and AST levels, histopathological damage, aggravated oxidative stress, and increased inflammatory responses. In the exercise model group, serum GSH decreased from 155.77 ± 9.32 to 71.64 ± 4.20 μmol/L, hepatic MDA increased from 3.80 ± 1.50 to 8.34 ± 1.58 nmol/mg protein, hepatic GSH decreased from 1.43 ± 0.05 to 1.00 ± 0.07 nmol/mg protein, and hepatic SOD decreased from 0.92 ± 0.56 to 0.33 ± 0.21 U/mg protein. Treatment with the tested total flavonoid preparation attenuated these alterations, with the medium-dose group showing the most consistent effects, including restoration of serum GSH to 135.84 ± 10.49 μmol/L, reduction of hepatic MDA to 4.07 ± 0.68 nmol/mg protein, increase of hepatic GSH to 1.35 ± 0.05 nmol/mg protein, and increase of hepatic SOD to 0.86 ± 0.07 U/mg protein. The preparation also reduced inflammatory mediator expression and modulated NF-κB–related signaling, as reflected by decreased p-IKKα and increased p-IκBα expression.

**Conclusion:**

The tested total flavonoid preparation attenuated exhaustive exercise–induced liver injury in mice. Its protective effects were associated with reduced oxidative stress, suppression of inflammatory responses, and modulation of NF-κB–related signaling, supporting its potential value in preventing exercise-related hepatic stress.

## 1 Introduction

With the rapid development of public fitness initiatives and competitive sports, the health impacts of high-intensity or excessive exercise have received increasing attention. Exhaustive exercise can trigger multisystem stress responses, among which the liver—an essential organ for energy metabolism and biotransformation—is particularly vulnerable to metabolic disturbance and stress-related injury (Thirupathi and Pinho, 2018; Egan and Zierath, 2013). Under conditions of high-intensity or prolonged exercise, hepatic glycogen stores are rapidly consumed. This glycogen-depleted state reflects increased energy demand and greater reliance on mitochondrial oxidative metabolism; under such conditions, increased oxygen flux through the electron transport chain may enhance electron leak and ROS formation, thereby promoting oxidative stress and hepatocellular injury (Powers et al., 2016; Zhao et al., 2019; Nolfi-Donegan et al., 2020).

Oxidative stress is considered one of the major pathological bases of exercise-induced liver injury. Excessive ROS generation promotes lipid peroxidation, resulting in elevated malondialdehyde (MDA) levels, while simultaneously impairing endogenous antioxidant defense systems, including superoxide dismutase (SOD) and glutathione (GSH) (He and Zuo, 2015). Persistent oxidative stress can further activate inflammation-related signaling pathways and stimulate the release of pro-inflammatory mediators, thereby forming a self-amplifying cycle between oxidative stress and inflammation that exacerbates hepatic injury (Mittal et al., 2014). Inflammatory responses also play a critical role in exercise-induced liver damage. Previous studies have shown that high-intensity exercise markedly upregulates the expression of pro-inflammatory cytokines such as tumor necrosis factor-α (TNF-α), interleukin-1β (IL-1β), and interleukin-6 (IL-6) (Suzuki, 2019). These cytokines can further induce the expression of inflammation-related proteins, including inducible nitric oxide synthase (iNOS), cyclooxygenase-2 (COX-2), and intercellular adhesion molecule-1 (ICAM-1), thereby aggravating hepatic inflammation and tissue injury. The nuclear factor-κB (NF-κB) pathway is widely recognized as a central regulatory link between oxidative stress and inflammation, and its aberrant activation has been observed in various models of hepatic inflammation and injury (Luedde and Schwabe, 2011; Sun, 2017). Accordingly, in addition to inflammatory cytokines, we examined IKKα/p-IKKα and IκBα/p-IκBα as indicators of NF-κB–related signaling status, together with iNOS, COX-2, and ICAM-1 as inflammation-related effector proteins. Among these markers, iNOS and COX-2 reflect pro-inflammatory effector activation, ICAM-1 is associated with leukocyte adhesion and inflammatory infiltration, and IKK/IκB phosphorylation reflects the activation status of NF-κB–related signaling.

In recent years, increasing attention has been paid to the potential value of traditional Chinese medicines and their bioactive metabolites in the prevention of exercise-related injury. *Puerariae Lobatae Radix* is a widely used botanical drug rich in flavonoid metabolites, including isoflavone-type metabolites such as puerarin, and has been associated with antioxidant, anti-inflammatory, and metabolic regulatory activities in previous pharmacological studies. Recent studies have shown that *Puerariae Lobatae Radix* and its major metabolites exert protective effects in models of metabolic disorders, cardiovascular diseases, and inflammation-related conditions (Zhou et al., 2014; Du et al., 2024; Jeon et al., 2020). However, the protective effects of the tested total flavonoid preparation derived from *Puerariae Lobatae Radix* against exhaustive exercise–induced liver injury, as well as its association with oxidative stress and inflammation-related signaling, remain insufficiently characterized.

Therefore, the present study employed a mouse model of liver injury induced by exhaustive swimming to systematically evaluate the protective effects of the tested total flavonoid preparation derived from *Puerariae Lobatae Radix* against exercise-induced hepatic damage and to further explore its potential association with oxidative stress, inflammatory responses, and NF-κB–related signaling.

## 2 Materials and methods

### 2.1 Investigational preparation and source information

The investigated material was a commercial finished pharmaceutical product, Getong Tongluo Jiaonang, manufactured by Anhui Jiufang Pharmaceutical Co., Ltd (Hefei, Anhui, China). The product specification was 0.25 g per capsule, the batch number used in this study was 181,002, and the labeled content of total flavonoids was ≥60%. According to the public product labeling and national drug standard, the product contains total flavonoids derived from *Puerariae Lobatae Radix*. The botanical drug source of *Puerariae Lobatae Radix* is recorded in the Chinese Pharmacopoeia as the dried root of *Pueraria lobata* (Willd.) Ohwi [Fabaceae]. Taxonomic resources such as Plants of the World Online currently treat this taxon within the *Pueraria montana* var. *lobata* complex. To avoid ambiguity and to remain consistent with pharmacopoeial usage, the botanical drug name *Puerariae Lobatae Radix* is used throughout this manuscript (Chinese Pharmacopoeia Commission, 2020).

The approved product standard for Getong Tongluo Jiaonang includes identity and quality-control information for the preparation, including thin-layer chromatographic identification and HPLC-based characterization using puerarin as a reference marker. Before administration, the capsule contents were freshly suspended in saline to the required concentrations (National Medical Products Administration, 2021).

Because the investigated material was a marketed finished pharmaceutical product rather than a laboratory-prepared extract or a batch prepared directly from freshly collected raw botanical material, source-level information typically reported for self-prepared botanical extracts—such as voucher deposition of the original raw material, drug–extract ratio, and full industrial extraction-process parameters—was not available to the authors for the specific batch used in this study. Therefore, all verifiable product-level information available from the product labeling and public national drug-standard documentation has been provided.

### 2.2 Animals

Forty-eight healthy adult male BALB/c mice (18–20 g) were obtained from the Experimental Animal Center of Anhui Medical University. Animals were housed under standard laboratory conditions (22 °C ± 2 °C, 50%–60% humidity, 12 h light/dark cycle) with free access to food and water and were acclimatized for 1 week before experiments. All experimental procedures were conducted in accordance with institutional guidelines for animal care and use.

### 2.3 Reagents

Commercial assay kits for ALT, AST, MDA, SOD, and reduced GSH were obtained from Nanjing Jiancheng Bioengineering Institute (Nanjing, China). ELISA kits for IL-1β (catalog No. JYM0531MO), IL-6 (catalog No. JYM0012MO), and TNF-α (catalog No. JYM0218MO) were purchased from Wuhan GeneBeauty Biotechnology Co., Ltd. (Wuhan, China). Antibodies against iNOS (catalog No. BS90715), COX-2 (catalog No. BS1076), IKKα (catalog No. BS1184), p-IKKα (catalog No. BS4100), IκBα (catalog No. BS90693), and p-IκBα (catalog No. BS4150) were obtained from Nanjing Biorbyt Biotechnology Co., Ltd. (Nanjing, China). The ICAM-1 antibody (catalog No. bs-0608R) was obtained from Bioss Biotechnology Co., Ltd. (Beijing, China).

### 2.4 Experimental design and drug administration

Mice with comparable body weights were manually allocated in equal numbers to six groups by different researchers: normal control (NC), exercise model (EM), silymarin-treated (SIL, 200 mg/kg/day), low-dose total flavonoid preparation (L-TF, 20 mg/kg/day), medium-dose total flavonoid preparation (M-TF, 50 mg/kg/day), and high-dose total flavonoid preparation (H-TF, 100 mg/kg/day). The NC and EM groups received an equal volume of vehicle (saline), whereas the remaining groups received the indicated treatments by oral gavage once daily for 4 weeks.

The dose range of the tested total flavonoid preparation (20, 50, and 100 mg/kg/day) was selected with reference to previous *in vivo* studies of total flavonoids from *Pueraria* and our related study using the same commercial preparation, in which a comparable low-/medium-/high-dose regimen showed biological activity. The silymarin dose (200 mg/kg/day) was selected as a commonly used hepatoprotective positive-control dose in rodent models of liver injury (Chen et al., 2023; Tsai et al., 2008; Sahin et al., 2020).

### 2.5 Exhaustive swimming protocol

During the final 10 days of treatment, all groups except the NC group underwent swimming training for 1 h/day between 9:00 and 11:00 a.m. in a temperature-controlled tank maintained at 30 °C ± 2 °C. After the final administration, mice were fasted for 12 h and subjected to an exhaustive swimming test on the last experimental day. Exhaustion was defined as continuous sinking and inability to return to the water surface within 10 s, upon which the mouse was immediately removed and the test was terminated. This criterion was adopted based on previous studies using exhaustive swimming models (Lima et al., 2013).

### 2.6 Sample collection

Immediately after exhaustive swimming, mice were euthanized and blood samples were collected via orbital puncture. Serum was separated by centrifugation. Livers were rapidly excised, rinsed, blotted dry, and weighed. Portions of liver tissue were fixed in 4% paraformaldehyde for histological and immunohistochemical analyses, while the remaining tissues were snap-frozen in liquid nitrogen and stored at −80 °C for biochemical assays, Western blotting, and RT-qPCR. The liver index was calculated as liver weight/body weight. Histopathological and immunohistochemical evaluations were performed in a blinded manner.

### 2.7 Histopathological examination

Fixed liver tissues were dehydrated, paraffin-embedded, sectioned at 4 μm thickness, and stained with hematoxylin and eosin (H&E). Histopathological changes were examined under a light microscope. Liver injury was evaluated with reference to the following morphological criteria: hepatocellular swelling, vacuolar degeneration, disorganization of hepatic cords, and inflammatory cell infiltration. For descriptive grading, the severity of each feature was interpreted as absent, mild, moderate, or severe in representative sections from each group. Hepatocellular swelling was assessed according to the extent of cellular enlargement and cytoplasmic pallor; vacuolar degeneration was assessed according to the presence and distribution of cytoplasmic vacuoles; disorganization of hepatic cords was assessed according to the loss of normal cord-like arrangement of hepatocytes; and inflammatory cell infiltration was assessed according to the extent and density of infiltrating inflammatory cells. Mild changes indicated focal or limited lesions with largely preserved architecture, moderate changes indicated more evident lesions involving a broader area with partial structural disturbance, and severe changes indicated extensive or diffuse lesions with marked structural disorganization and/or prominent inflammatory infiltration.

### 2.8 Biochemical analysis

Serum ALT, AST, and GSH levels were determined using commercial assay kits (catalog Nos. C009-2-1, C010-2-1, and A005-1-2, respectively; Nanjing Jiancheng Bioengineering Institute, Nanjing, China) according to the manufacturers’ instructions. Hepatic MDA content and SOD and GSH activities were measured using the corresponding commercial assay kits (catalog Nos. A003-1-2, A001-3-2, and A005-1-2, respectively; Nanjing Jiancheng Bioengineering Institute, Nanjing, China) following the manufacturers’ protocols. Hepatic biochemical parameters were normalized to protein content.

### 2.9 Measurement of inflammatory cytokines

Levels of IL-1β, IL-6, and TNF-α in serum and liver homogenate supernatants were quantified using commercial ELISA kits (catalog Nos. JYM0531MO, JYM0012MO, and JYM0218MO, respectively; Wuhan GeneBeauty Biotechnology Co., Ltd., Wuhan, China) according to the manufacturers’ instructions.

### 2.10 Immunohistochemistry

Paraffin-embedded liver sections were deparaffinized, rehydrated, and subjected to antigen retrieval, followed by incubation with primary antibodies against iNOS (catalog No. BS90715), COX-2 (catalog No. BS1076), and ICAM-1 (catalog No. bs-0608R). After incubation with the appropriate secondary antibodies, immunoreactivity was visualized and scanned using a slide scanning system. Immunohistochemical staining was semi-quantitatively analyzed using ImageJ/Fiji software. For each marker, positive staining was evaluated in at least three representative microscopic fields per section under the same acquisition conditions. The integrated optical density (IOD) and positive staining area were measured, and the average optical density (AOD) was calculated as IOD/area for comparative analysis (Schindelin et al., 2012).

### 2.11 Western blot analysis

Total protein was extracted from liver tissues and quantified. Equal amounts of protein were separated by SDS-PAGE and transferred onto PVDF membranes. After blocking with 5% BSA, membranes were incubated overnight at 4 °C with primary antibodies against IKKα (catalog No. BS1184), p-IKKα (catalog No. BS4100), IκBα (catalog No. BS90693), and p-IκBα (catalog No. BS4150), followed by incubation with the appropriate secondary antibodies. Protein bands were visualized using an ECL detection system. Band intensities were quantified by densitometric analysis using ImageJ software. β-Actin was used as the internal control, and the relative expression of each target protein was normalized to β-actin. The experiments were independently repeated at least three times, and representative blots are shown.

### 2.12 RT-qPCR analysis

Total RNA was extracted from liver tissues and reverse-transcribed into cDNA. The mRNA expression levels of IL-1β, IL-6, and TNF-α were determined by quantitative real-time PCR using β-actin as the internal reference gene. The primer sequences were as follows: IL-1β forward, 5′-CTT​CAG​GCA​GGC​AGT​ATC​ACT​C-3′, and reverse, 5′-TTG​TTG​TTC​ATC​TCG​GAG​CC-3′; IL-6 forward, 5′-CCA​CGG​CCT​TCC​CTA​CTT​C-3′, and reverse, 5′-CTC​ATT​TCC​ACG​ATT​TCC​CAG-3′; TNF-α forward, 5′-CAT​CTT​CTC​AAA​ATT​CGA​GTG​ACA​A-3′, and reverse, 5′-TGG​GAG​TAG​ACA​AGG​TAC​AAC​CC-3′.

### 2.13 Statistical analysis

Data are presented as mean ± SD. Statistical analyses were performed using SPSS 16.0 software. Comparisons among multiple groups were performed using one-way analysis of variance (ANOVA) followed by Tukey’s *post hoc* test. A value of P < 0.05 was considered statistically significant. “*” indicates P < 0.05 versus the NC group, and “#” indicates P < 0.05 versus the EM group.

## 3 Results

### 3.1 Total flavonoids from *Puerariae Lobatae Radix* attenuate exhaustive exercise–induced liver injury and improve hepatic function

**FIGURE 1:**
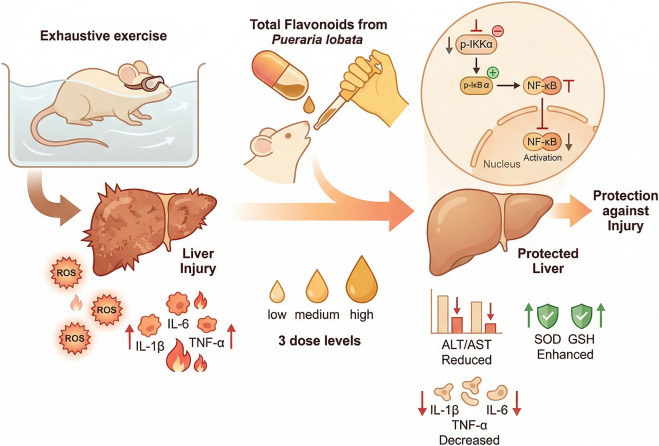
Experimental design of the study. Schematic illustration of the experimental protocol. Mice were manually allocated in equal numbers to six groups by different researchers and received daily administration for 4 weeks. During the final 10 days, exhaustive swimming training was performed, followed by an exhaustive swimming test on the last experimental day. Liver function, histopathology, oxidative stress, inflammatory responses, and NF-κB–related signaling were subsequently evaluated.

**FIGURE 2:**
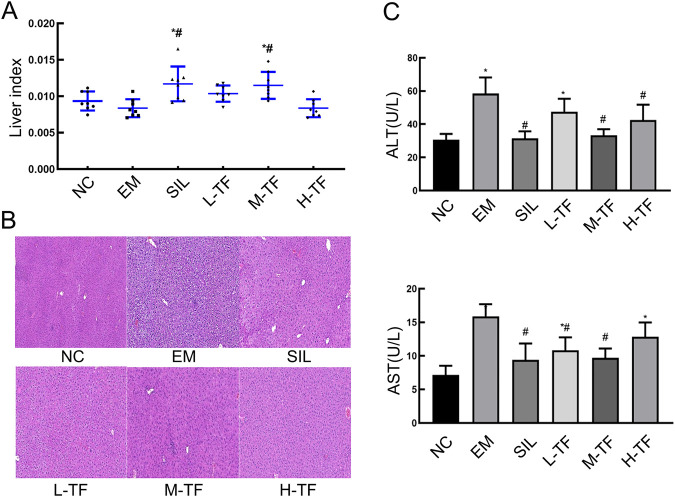
Effects of total flavonoids from *Puerariae Lobatae Radix* on liver injury and hepatic function in mice subjected to exhaustive exercise. **(A)** Liver index of mice in different groups. **(B)** Representative hematoxylin and eosin (H&E)–stained liver sections showing histopathological changes (scale bar = 100 μm). **(C)** Serum ALT and AST levels. Data are presented as mean ± SD (n = 8). *P < 0.05 vs. the normal control (NC) group; #P < 0.05 vs. the exercise model (EM) group.

The liver index and liver function–related parameters are commonly used indicators for evaluating hepatic status and injury severity. To initially assess the effects of exhaustive exercise on the liver and the intervention of total flavonoids from *Puerariae Lobatae Radix* (experimental design shown in Figure 1), liver index, histopathological alterations, and serum ALT and AST levels were examined. As shown in Figure 2A, the liver index in the exercise model group exhibited a decreasing trend compared with the normal control group, although the difference was not statistically significant. In contrast, the silymarin-treated group and the medium-dose total flavonoids group showed a significant increase in liver index compared with the exercise model group (P < 0.05), whereas no significant changes were observed in the low- or high-dose groups.

Histopathological examination by H&E staining is shown in Figure 2B. Liver sections from the normal control group displayed intact hepatic architecture with regularly arranged hepatocytes and clearly defined nuclei. In the exercise model group, pronounced pathological alterations were observed, including hepatocellular swelling, increased vacuolar degeneration, disorganization of hepatic cords, and inflammatory cell infiltration. Compared with the exercise model group, liver injury was markedly alleviated in the silymarin-treated and medium-dose total flavonoids groups, with relatively preserved hepatic architecture and a substantial reduction in vacuolar degeneration. The low- and high-dose groups also exhibited partial improvement, although focal hepatocellular degeneration remained evident.

To further evaluate hepatic functional changes, serum ALT and AST levels were measured (Figure 2C). Compared with the normal control group, the exercise model group showed significantly elevated serum ALT and AST levels (P < 0.05), indicating apparent liver dysfunction induced by exhaustive exercise. ALT increased from roughly 30 U/L in the normal control group to nearly 60 U/L in the exercise model group, whereas AST increased from about 7 U/L to around 16 U/L. In contrast, the medium-dose total flavonoid preparation brought both indicators back toward the normal range, whereas the high-dose group showed only partial improvement, suggesting that the medium-dose regimen produced the most consistent hepatoprotective effect.

### 3.2 Total flavonoids from *Puerariae Lobatae Radix* alleviate exhaustive exercise–induced hepatic oxidative stress

Given that exhaustive exercise can induce excessive production of reactive oxygen species and trigger oxidative stress, oxidative stress-related parameters were further assessed to evaluate the regulatory effects of total flavonoids on exercise-induced oxidative damage. Serum GSH levels and hepatic MDA, GSH, and SOD levels are summarized in Table 1. Compared with the normal control group, the exercise model group exhibited a significant reduction in serum GSH levels, a marked increase in hepatic MDA content, and significant decreases in hepatic GSH and SOD levels (P < 0.05). Specifically, serum GSH decreased from 155.77 ± 9.32 μmol/L in the NC group to 71.64 ± 4.20 μmol/L in the EM group, whereas hepatic MDA increased from 3.80 ± 1.50 to 8.34 ± 1.58 nmol/mg protein, indicating a pronounced oxidative stress state induced by exhaustive exercise. In comparison with the exercise model group, the silymarin-treated and medium-dose total flavonoid preparation groups showed significant improvement in these parameters (P < 0.05). In the medium-dose group, serum GSH recovered to 135.84 ± 10.49 μmol/L, hepatic MDA decreased to 4.07 ± 0.68 nmol/mg protein, hepatic GSH increased to 1.35 ± 0.05 nmol/mg protein, and hepatic SOD increased to 0.86 ± 0.07 U/mg protein.

**TABLE 1:** Effects of total flavonoids from *Puerariae Lobatae Radix* on oxidative stress–related parameters in serum and liver of mice.

Group	Serum GSH (µmol/L)	Hepatic MDA (nmol/mg protein)	Hepatic GSH (nmol/mg protein)	Hepatic SOD (U/mg protein)
NC	155.77 ± 9.32	3.80 ± 1.50	1.43 ± 0.05	0.92 ± 0.56
EM	71.64 ± 4.20*	8.34 ± 1.58*	1.00 ± 0.07*	0.33 ± 0.21*
SIL	131.65 ± 7.25*^,#^	4.00 ± 1.17^#^	1.28 ± 0.04*^,#^	0.81 ± 0.16#
L-TF	85.90 ± 15.87*	5.33 ± 0.53^#^	1.14 ± 0.10*^,#^	0.48 ± 0.03*
M-TF	135.84 ± 10.49*^,#^	4.07 ± 0.68^#^	1.35 ± 0.05^#^	0.86 ± 0.07#
H-TF	94.82 ± 8.70*^,#^	5.90 ± 0.63*^,#^	1.21 ± 0.05*^,#^	0.57 ± 0.03*

Abbreviations: NC, normal control; EM, exercise model; SIL, silymarin; L-TF, low-dose total flavonoids; M-TF, medium-dose total flavonoids; H-TF, high-dose total flavonoids.

Statistical analysis:

Data are presented as mean ± SD (n = 8).

*P < 0.05 vs. NC group; #P < 0.05 vs. EM group.

### 3.3 Total flavonoids from *Puerariae Lobatae Radix* suppress systemic and hepatic inflammatory responses induced by exhaustive exercise

**FIGURE 3:**
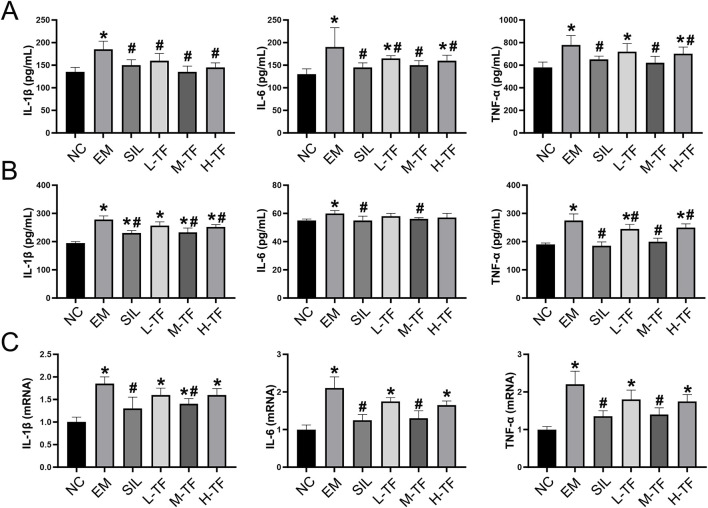
Effects of total flavonoids from *Puerariae Lobatae Radix* on systemic and hepatic inflammatory responses induced by exhaustive exercise. **(A)** Serum levels of IL-1β, IL-6, and TNF-α. **(B)** Protein levels of IL-1β, IL-6, and TNF-α in liver tissue. **(C)** Relative mRNA expression levels of IL-1β, IL-6, and TNF-α in liver tissue. Data are presented as mean ± SD (n = 8). *P < 0.05 vs. the NC group; #P < 0.05 vs. the EM group.

Oxidative stress is often accompanied by activation of inflammatory responses. To determine whether exhaustive exercise-induced liver injury was associated with inflammatory activation, the expression levels of IL-1β, IL-6, and TNF-α in serum and liver tissue were examined (Figures 3A,B). Compared with the normal control group, the exercise model group showed significantly elevated levels of IL-1β, IL-6, and TNF-α in both serum and liver tissue (P < 0.05). Treatment with silymarin or the tested total flavonoid preparation reduced these inflammatory cytokines to varying degrees, with the medium-dose group showing the most consistent reduction (P < 0.05).

The mRNA expression levels of IL-1β, IL-6, and TNF-α in liver tissue were further analyzed (Figure 3C). Consistent with the protein data, hepatic mRNA expression of these cytokines was significantly upregulated in the exercise model group compared with the normal control group (P < 0.05), whereas treatment with the tested total flavonoid preparation, particularly at the medium dose, markedly reduced their expression (P < 0.05).

### 3.4 Total flavonoids from *Puerariae Lobatae Radix* downregulate hepatic inflammation-related proteins and modulate NF-κB signaling activation

**FIGURE 4:**
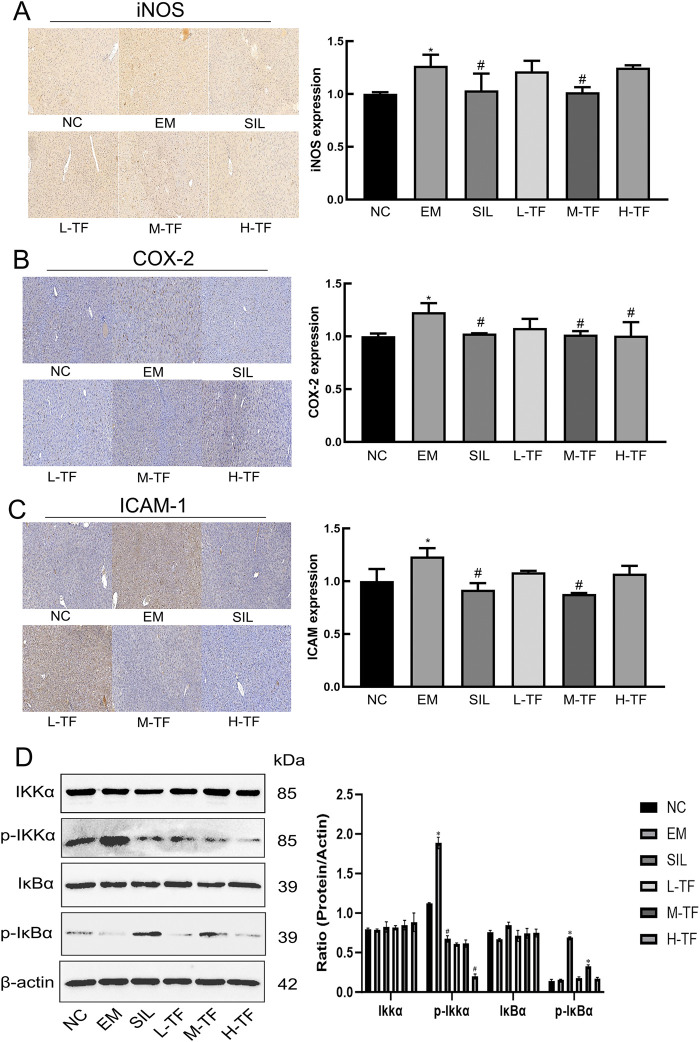
Effects of total flavonoids from *Puerariae Lobatae Radix* on inflammation-related protein expression and NF-κB signaling activation in the liver. **(A–C)** Representative immunohistochemical staining of iNOS, COX-2, and ICAM-1 in liver tissue (scale bar = 100 μm). *P < 0.05 vs. the NC group; #P < 0.05 vs. the EM group. **(D)** Representative western blots of IKKα, p-IKKα, IκBα, and p-IκBα in liver tissue. β-Actin was used as the internal control. The experiments were independently repeated at least three times with similar trends. *P < 0.05 vs. the NC group; #P < 0.05 vs. the EM group.

To further evaluate inflammation-related effector proteins and NF-κB-related signaling in liver tissue, the expression of iNOS, COX-2, and ICAM-1 was examined by immunohistochemistry, together with the protein levels of IKKα, p-IKKα, IκBα, and p-IκBα by Western blotting (Figure 4).

Immunohistochemical analysis (Figures 4A–C) showed that the expression of iNOS, COX-2, and ICAM-1 was markedly increased in the exercise model group compared with the normal control group. In contrast, silymarin treatment and the medium-dose total flavonoid preparation markedly reduced the expression of these proteins, whereas the low- and high-dose groups showed only partial attenuation.

Western blot analysis (Figure 4D) showed that the relative level of p-IKKα was increased and that of p-IκBα was decreased in the exercise model group compared with the normal control group. Treatment with the tested total flavonoid preparation reversed these changes, with the medium-dose group showing the most consistent effect. No obvious differences were observed in total IKKα or IκBα expression among groups. These findings indicate that the protective effect of the tested preparation was associated with modulation of NF-κB-related signaling rather than changes in total protein abundance.

## 4 Discussion

Exhaustive exercise–induced liver injury is often not readily identifiable at an early stage because of the liver’s substantial compensatory capacity. Nevertheless, the metabolic burden and stress responses triggered by high-intensity exercise can accumulate and eventually present as hepatic functional abnormalities accompanied by histopathological damage. Using an exhaustive swimming model, the present study evaluated hepatic function, histological alterations, oxidative stress, inflammatory responses, and NF-κB–related signaling proteins. The results showed that exhaustive exercise induced clear hepatic stress and injury, whereas intervention with the tested total flavonoid preparation produced multi-level improvement, supporting a hepatoprotective effect.

In the exercise model group, elevations in serum ALT and AST were accompanied by hepatocellular swelling, vacuolar degeneration, and inflammatory infiltration, consistent with an acute hepatic stress phenotype. Recent studies have highlighted the contribution of immune-cell recruitment to exercise-induced tissue injury. For example, neutrophil depletion has been reported to attenuate hepatic stress-marker elevation after exhaustive exercise, indicating that inflammatory infiltration is not merely a bystander phenomenon (Mizokami and Suzuki, 2023). Similarly, neutrophils and macrophages have been implicated in multi-organ injury after exhaustive exercise through enhanced ROS production and inflammatory mediator release (Mizokami and Suzuki, 2024). In the present study, the tested preparation reduced serum transaminase levels and alleviated histological damage, indicating a protective effect at both functional and structural levels.

Oxidative stress is widely recognized as a central pathological basis of exhaustive exercise–induced liver injury. In the present study, the exercise model group showed increased hepatic MDA levels together with reduced serum GSH, hepatic GSH, and SOD, indicating enhanced lipid peroxidation and impaired antioxidant defense. These alterations were substantially improved after treatment, particularly in the medium-dose group. These findings are consistent with antioxidant and hepatoprotective effects reported for *Puerariae Lobatae Radix*–related preparations and puerarin in other liver injury models, and support the view that reinforcement of endogenous antioxidant capacity is an important component of the observed protection (Dong et al., 2024; Li et al., 2024; He et al., 2024). These findings are also in line with recent studies showing that natural-product interventions can alleviate liver injury through coordinated suppression of oxidative stress and inflammatory signaling, including reports on Chlorella vulgaris, Opuntia ficus-indica, beta-caryophyllene, and resatorvid/alpha-lipoic acid in experimental liver injury models (Melebary, 2025; Hafeez et al., 2024; Yilma et al., 2025; Hatipoğlu et al., 2024; Saeed et al., 2023).

Inflammatory activation represented another major feature of the model. IL-1β, IL-6, and TNF-α were significantly increased at both protein and mRNA levels in serum and liver tissue after exhaustive exercise, indicating coordinated systemic and local inflammatory activation. Treatment reduced these cytokines at both levels, especially in the medium-dose group, supporting an anti-inflammatory effect of the tested preparation. Together with the concurrent improvement in oxidative stress parameters, these findings are consistent with attenuation of the reciprocal amplification between oxidative stress and inflammation.

At the tissue-effector level, iNOS and COX-2 represent sustained pro-inflammatory activation, whereas ICAM-1 is more closely associated with leukocyte adhesion and inflammatory infiltration. In the present study, exhaustive exercise markedly increased the expression of iNOS, COX-2, and ICAM-1, whereas treatment reduced their expression (Heo et al., 2023; Kim and Lee, 2025; Zhili et al., 2025). At the signaling level, the exercise model group showed increased p-IKKα and decreased p-IκBα expression, while treatment reversed these changes without significantly altering total IKKα or IκBα levels. This phosphorylation-dominated pattern is consistent with modulation of NF-κB–related signaling rather than a change in total protein abundance (Zhang et al., 2023; Yang et al., 2024). However, because nuclear NF-κB translocation and downstream target-gene activation were not directly measured, these data should be interpreted as associative rather than definitive mechanistic evidence.

Notably, the medium-dose group showed more consistent protection than the high-dose group. This pattern suggests a non-linear dose–response relationship, which is not uncommon in studies of plant-derived preparations. Possible explanations include absorption saturation, accelerated metabolism, interactions among multiple metabolites, or an additional metabolic burden at higher doses. Accordingly, the present results support an empirical conclusion that the medium-dose regimen provided the most stable overall benefit, whereas the basis of dose-dependent differences requires further pharmacokinetic and exposure-based investigation (Hao et al., 2024).

Several limitations should be acknowledged. First, the investigated material was a commercial finished total flavonoid preparation, and although product-level information such as batch number, labeled flavonoid content, and public standard-based identity/quality-control information could be verified, some source-level information typically required for fully characterized self-prepared botanical extracts, including voucher deposition of the original raw botanical material, drug–extract ratio, and detailed industrial extraction parameters, was not available for the specific batch used in this study. Second, direct assessment of NF-κB nuclear translocation or downstream target-gene activation was not performed. Third, hepatic glycogen depletion was not directly measured, and therefore the link between exercise-related energy depletion and oxidative liver injury remains inferential. Fourth, the specific animal model and experimental conditions limit direct clinical relevance and broader extrapolation of the present findings.

In conclusion, the present study provides multi-level evidence that the tested total flavonoid preparation attenuates exhaustive exercise–induced liver injury in mice. Its protective effects were associated with reduced oxidative stress, suppression of inflammatory responses, and modulation of NF-κB–related signaling. These findings support the potential value of this preparation in the prevention of exercise-related hepatic stress and provide a basis for further mechanistic and translational investigation.

## Data Availability

The original contributions presented in the study are included in the article/supplementary material, further inquiries can be directed to the corresponding author.
